# RatLab: an easy to use tool for place code simulations

**DOI:** 10.3389/fncom.2013.00104

**Published:** 2013-07-29

**Authors:** Fabian Schönfeld, Laurenz Wiskott

**Affiliations:** Theory of Neural Systems Group, Institute for Neural Computation, Ruhr University BochumBochum, Germany

**Keywords:** simulation, place cells, head direction cells, Slow Feature Analysis, MDP, CUDA

## Abstract

In this paper we present the RatLab toolkit, a software framework designed to set up and simulate a wide range of studies targeting the encoding of space in rats. It provides open access to our modeling approach to establish place and head direction cells within unknown environments and it offers a set of parameters to allow for the easy construction of a variety of enclosures for a virtual rat as well as controlling its movement pattern over the course of experiments. Once a spatial code is formed RatLab can be used to modify aspects of the enclosure or movement pattern and plot the effect of such modifications on the spatial representation, i.e., place and head direction cell activity. The simulation is based on a hierarchical Slow Feature Analysis (SFA) network that has been shown before to establish a spatial encoding of new environments using visual input data only. RatLab encapsulates such a network, generates the visual training data, and performs all sampling automatically—with each of these stages being further configurable by the user. RatLab was written with the intention to make our SFA model more accessible to the community and to that end features a range of elements to allow for experimentation with the model without the need for specific programming skills.

## Introduction

When navigating in known environments or exploring new ones, rats display a distinct spatial code in their hippocampus. It consists of the firing patterns of grid cells in the entorhinal cortex (Hafting et al., 2005), place cells in the CA3 and CA1 areas (O'Keefe and Dostrovsky, 1971), and head direction cells in the subiculum (Taube et al., 1990). During development it can be observed that head direction cells reach adult-like performance as soon as young rats open their eyes. Place cells take until age P16–P17 to reach adult level, while grid cell arrive there at age P20–P21 (Langston et al., 2010; Willis et al., 2010). When rats enter and subsequently move within a novel environment, their head direction cells as well as the grid cell system anchor themselves quickly to the available visual cues (Taube and Burton, 1995). Place cells on the other hand require about 10 min of exploration time—during which single place fields may change considerably—before the whole system settles into a stable state (Wilson and McNaughton, 1994; Frank et al., 2004). This encoding of place is persistent and will from then on be available in the associated environment without the need for further training (Thompson and Best, 1990).

It is currently unknown how exactly place fields form during the initial exploration phase of an environment. It seems clear, however, that visual information plays an essential role in this process as the spatial representation in the rat is usually bound to salient visual cues in the environment (Knierim et al., 1995; Goodridge et al., 1998). It is therefore a straightforward question to ask whether we can construct a model that is primarily built on visual input and able to produce signals featuring the same peaks in activity that we observe in place field recordings. (Franzius and Wiskott, 2007) have shown how this can be done with a hierarchical network of nodes implementing the Slow Feature Analysis (SFA) algorithm and a final sparse coding step. This model takes a sequence of images from an exploration phase as its input for training and afterwards can be sampled over the environment where it displays the expected firing properties of different place cells.

One motivation for RatLab is the attempt to provide a user friendly access point to using this model in further experiments. Although the core network is described in detail in Franzius and Wiskott (2007), replicating the software can be time consuming—and more importantly—largely excludes scientists from other fields without extended programming experience. RatLab is a Python based software tool designed to bypass these issues and allow the easy setup of a wide range of navigational experiments. It includes the same hierarchical SFA network as described in Franzius and Wiskott (2007) and offers the additional functionality to set up user defined environments, configure the statistical behavior of the virtual rodent, and produce a multitude of place code plots.

The software simulates random exploration experiments where a rat is foraging for randomly thrown food pellets. In such tasks the rat has no specific goal other than to keep moving in order to passively generate a stable place code to be observed. Once established this representation can be examined while being subjected to various manipulations of the environment. Examples include stretching a box in a certain direction as done in O'Keefe and Burgess (1996) or morphing an enclosure from a circular environment to a rectangular one as presented in Wills et al. (2005). Other experiments are concerned with the binding of the place code to prominent visual cues in the environment, where it can be observed that the spatial representation of the place code rotates with the rotation of notable cues (Knierim et al., 1995; Goodridge et al., 1998). All of these experiments merely require the random foraging of the rat without the need of an explicit goal for the rat to reach—as would be the case in experimental setups like the Morris Water Maze (Morris, 1981). In addition to experiments based on random movement the user may also define a list of navigation points which are repeatedly traversed in the given order. This can simulate a rat that was trained to follow a certain path within a maze—for example to always turn left at the crossroad of a T-maze, or to always run up and down a single corridor. Studies like these can aim at the loss of directional invariance of the place code, i.e., the examination of place fields that fire only when being traversed in a certain direction as shown in McNaughton et al. (1983); O'Keefe and Recce (2004) and reproducible by RatLab.

The following part describes how simulations can be set up and which parameters and options are available. While this section also briefly describes the structure of the framework, the technical details are summarized in the third part of the text, including a short overview of the theoretical principle at the heart of the model.

## Running a simulation

**Figure 1:**
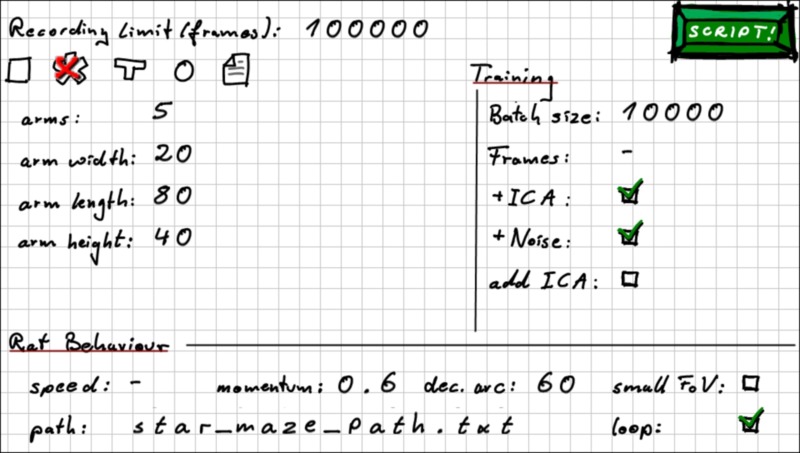
**RatLab GUI mask**. To facilitate the quick setup of basic experiments RatLab offers this GUI mask to create a full simulation script from the presented parameter set. Note that this is primarily a learning tool to help users to ultimately write elaborate scripts. The values shown in this example yield the script shown in the second part of this section.

In order to allow for easier access to RatLab, the software comes with several elements to help users familiarize themselves with the framework. This includes an online tutorial, a short guide to the packages used by Ratlab, and a script to check whether all of them are available to Python on a given PC. The RatLab framework itself consists of four core modules that are run in succession, and consequently a full simulation is run by a script containing four calls to this “pipeline” of modules. In order to not burden beginner users with the process of writing a shell script, however, the RatLab framework includes a hand drawn input mask that looks like a plain sheet of paper with most of the basic parameters written down on it (Figure 1). From these values a basic shell script can be generated that executes a complete RatLab simulation. These shell scripts can also be studied together with the RatLab documentation in order to make use of the full set of the parameters available to the software. The documentation also contains several examples to assist in this and is available by calling any of the core modules with the “-help” parameter.

The available parameters of the RatLab framework are either part of the more technical background (like the nearest distance the virtual rodent will be able to walk up to a wall) or are part of the setup options for an actual experiment (like placed obstacles within the enclosure). All of the former, more technical, values will typically stay the same over several, if not all, simulations and are therefore collected in a central configuration file that can be edited with any text editor. The experimental setup parameters on the other hand are implemented as arguments to be used when calling RatLab's core Python modules. In this way it is possible to read a simulation script and clearly be able to tell what the experiment will look like.

**Figure 2:**
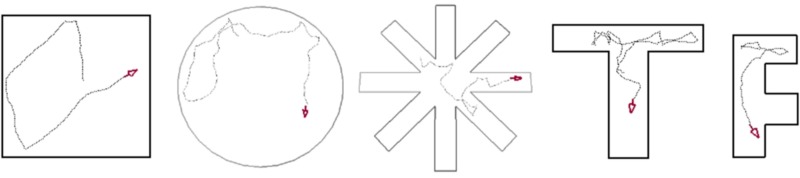
**RatLab enclosure templates**. RatLab offers a selection of configurable templates for the most common experimental setups. Parameters like the radius for a circle maze, or the number of arms for a star maze can all be chosen freely. RatLab constructs and renders the specified environment and thereby produces the training data for our model. It also offers the option to define a fully custom environment in case none of the presets fits to a desired experiment.

Most setup parameters belong to the first of these core modules (ratlab.py) which implements the construction and rendering of the environment as well as the control of the virtual rodent over the course of the simulation. The available options to this module include the number of steps the simulation will run, whether or not visual data should be recorded, and if so, whether it should be recorded in color, grayscale, or both. In terms of setting up the enclosure for the experiment the user may choose between several parameterized presets (see Figure 2) or specify a file describing a completely user-generated environment. In case of the latter it is only required to define the start and end points of the desired wall segments (optionally using user generated textures) in a common.txt file. If indeed a custom maze is used, RatLab can also be executed in “wallcheck” mode which renders two overview images of the environment—one with a raster overlay and one without—to enable a user to quickly check the floor plan geometry for gaps. For further customization rectangular boxes can be placed as additional obstacles within the chosen environments. The textures for all wall segments and obstacles can optionally be triggered to be the same or cycle through the available set of image files. These are part of the file structure of the framework and thus easy to replace, extend, or modify.

The virtual rat cannot be controlled directly. Its behavior is controlled instead by a collection of parameters that define its movement statistics over time. This includes the base speed of the rat, its momentum, and its maximal rotation per time step. The momentum value controls the curvature of path segments, with higher momentum values resulting in straighter paths. As mentioned in the introduction a distinct series of waypoints can also be specified to imitate a rat trained on a specific path. This is done by first rendering the blueprint of the environment with a coordinate system overlay via the “wallcheck” option. This image is then used to determine the distinct path coordinates and store them within a.txt file. Using the “path” parameter, the simulated rodent heads for each of the specified waypoints in sequential order. A noise parameter is included to create a more irregular movement along the path. The amount of this variation is an example of the values stored in the RatLab configuration file, as it is unlikely to change over experiments. It can also be specified whether the virtual rodent's position should be reset upon reaching the final waypoint or whether it should continue on to the first waypoint and thus run in a continuous loop. In addition to the parameters that focus on movement, it can also be specified whether the rat should use its full field of view (FoV) of 320 degrees (Hughes, 1979) or be restricted to a FoV of only 55 degrees. This smaller FoV option is provided to train the model with the narrower FoV of a digital camera, thus enabling RatLab to more easily work together with robots.

The script produced by accepting the values shown in Figure 1 is:

python ratlab.py record limit 100000 color star_maze 5 20 80 40 mom 0.6 arc 60 path
star_maze_path.txt loop
python convert.py
python train.py batch_size 10000 ICA noise
python sample.py - 1 32 all

**Figure 3:**
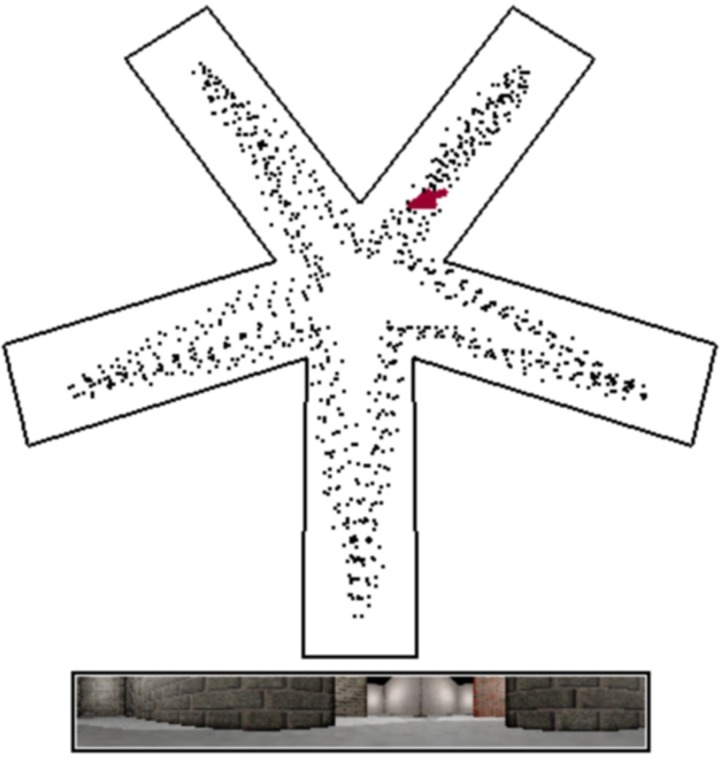
**RatLab simulation view**. By default RatLab shows an overhead view of the specified environment. The position and direction of the virtual rodent is indicated by a red arrow, and the path traversed so far by a white dotted line. Below this overview the current visual input to the rat is rendered. In this example the rat is also trained to visit each of the five arms in succession and does not randomly explore the environment. To speed up the runtime of simulations some elements of rendering RatLab frames can be switched off. Screenshots as shown here can be taken at any time during the simulation by pressing F12; the image is then labeled and stored in the working directory of the experiment. This simulation is based on the parameters shown in Figure 1.

This translates to running a simulation for 100.000 steps while recording the visual information both as color as well as grayscale images. The environment is a star maze consisting of five equidistantly positioned arms. The virtual rodent runs with a momentum of 0.6 and during a single time step cannot turn more than 60 degrees. It follows a path specified in the file ‘star_maze_path.txt’ and loops back to the first waypoint upon reaching the final one. Figure 3 shows a screenshot of RatLab running this particular setup. After running for the specified amount of time steps a hierarchical SFA network is trained with the recorded image data, using 10 batches of 10.000 frames at a time. The network contains an optional final step of ICA (independent component analysis) and adds additional noise to the information propagated through its different layers.

During a simulation a full set of place field plots is created and stored in a local folder. These fields can be checked for being directional dependent by comparing sampling plots where the rat/camera points in a distinct direction to plots that are averaged over eight different directions. All the necessary plots are automatically generated and uniquely labeled. After executing and studying basic scripts, users may then use the included documentation in order to learn and make use of the additional parameters not featured on the basic GUI input mask. This enables multi-stage experiments as well as adapting RatLab simulations to specific needs.

One final feature is to use RatLab to train the network in a generic way. By default, training data consists of images produced by traversing a particular environment and is being used to train a full hierarchical network. Generic training, on the other hand, uses a range of natural images as training data and merely trains the lower layers of the hierarchical network. The images used may be extracted from real life video streams, parts of movies, or any other image sequence containing the statistics of natural images and sufficient variety. This also includes sets of images recorded over several different RatLab environments. A network trained this way is able to extract low level features like edges and basic patterns. The remaining upper layers of the network are then trained within a single specific environment in order to learn a place code associated with this enclosure. This overall process has the benefit of creating a generalized base network that can be used in several environments by merely training the upper layer in each one of them. Since only one layer needs to be trained after generalized training, this also works faster than training a new complete network for each of several different environments. Note, however, that the model has no memory state, i.e., once trained in a second environment, the place code of a first environment is usually lost. To facilitate generalized training RatLab offers a tool to cut out the central parts of a series of images, thus providing a quick way to produce training data in the format required for RatLab.

Besides being a more efficient way to train networks for several environments, generalized training is also more analogous to the biological development of the visual system. The lower network layers are developed first and serve to detect basic features like edges. This generic functionality becomes fixed and is subsequently reused by the higher layers of the hierarchy in order to recognize higher level features in the visual input when learning a new environment.

## Model background

Understanding the technical details of RatLab is not required to make use of the toolkit or the model. However, as the complete package is freely available online, users are encouraged to modify or extend the framework as they see fit. Thus, the following two sections describe the theoretical foundation of our approach and the implementation details of the hierarchical model as it is used in the software.

### Slow feature analysis

Our approach to extract place field signals from training with visual data is based on SFA, an unsupervised learning algorithm described in detail in Wiskott and Sejnowski (2002). It is built around the assumption than meaningful information within an input stream of data varies slowly in time and aims at finding a way to combine incoming signals in a way as to yield output signals that satisfy this condition. More precisely, SFA takes a multidimensional input signal **x**(*t*) = [**x**_1_(*t*), **x**_2_(*t*),…, **x**_N_(*t*)]^T^ and finds a set of functions *g*_1_(**x**), *g*_2_(**x**),…, *g*_*k*_(**x**) such that each output signal *y*_*i*_(*t*): = *g*_*i*_[**x**(*t*)] features the least amount of variation over time possible, i.e.,

(1) $$\Delta (y_{i}):\;={\langle {\dot{y}}_{i}^{2}\rangle }_{t}\text{ is minimal}.$$

The slowest possible signal would always be a constant value that does not change over time and thus carries no information. In order to avoid this, as well as to force SFA to not yield the same slow signal twice, the set of output signals *y*_*i*_(*t*) further adheres to the following constraints:

(2) $$\text{Zero}\;\text{mean}:\;{\langle y_{i}\rangle }_{t}=0.$$

(3) $$\text{Unit}\;\text{variance}:\;{\langle y_{i}^{2}\rangle }_{t}=1.$$

(4) $$\text{Decorrelation}\;\text{and}\;\text{order}:\;\forall i<j,\;{\langle y_{i},y_{j}\rangle }_{t}=0.$$

The SFA algorithm works by using a (usually quadratic) expansion of the input signal data, whitening it, and computing the covariance matrix of both the actual signals as well as their first derivatives. By solving a generalized eigenvalue problem SFA acquires the eigenvectors of these matrices which hold the values that in turn serve as the coefficients in the functions *g*_*i*_(*x*) (cf. Berkes and Wiskott, 2005).

**Figure 4:**
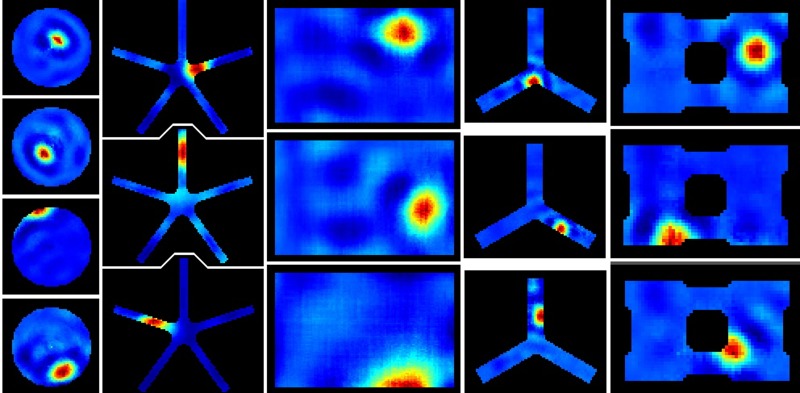
**RatLab place field results**. After training the generated SFA hierarchy is sampled over the learned environments. Here the resulting place fields of a variety of simulations are shown. From left to right: a basic circular enclosure, a radial arm maze featuring five arms, a rectangular box, a three-arm maze, and a rectangular enclosure filled with a variety of obstacles along the walls (visible as black dents at the edges of the plots) and one in the center. Note the five arm star maze plots depicting the results of the simulation that is defined in the script example of the text and shown running in Figure 3.

**Figure 5:**
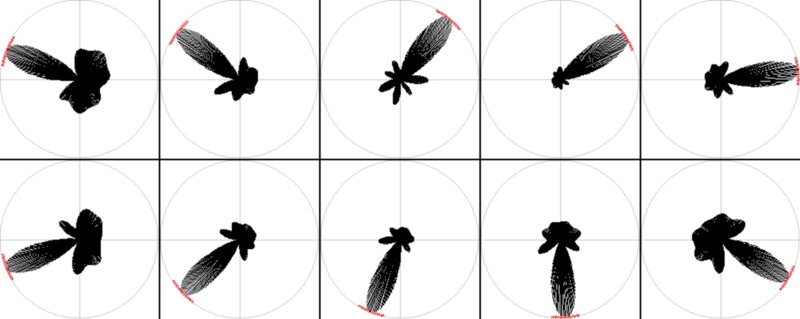
**RatLab head direction results**. The SFA hierarchy can also be trained to respond to the head direction of the agent, as shown in these samples.

When training SFA with the quickly varying visual input stream generated by RatLab a set of functions is found that allow to extract the more slowly varying location. After training this established spatial code is available in real time, i.e, the model requires only a single visual input frame in order to respond with place field firing at the current position. Note, however, that raw SFA output alone does not yet display the desired place field patterns. While the nonlinear computation of the SFA hierarchy does provide a mapping from visual input to distinct location information, it is only with an additional step of sparse coding that these output patterns form place fields as shown in Figure 4. When using RatLab this additional step is performed by ICA (independent component analysis), a linear transformation that rotates the raw SFA output to form a more sparse code that results in clear place fields. Whether ICA is attached to a SFA hierarchy in RatLab is determined by an “ICA” parameter in the Python script—it can be seen as a selected option in the RatLab GUI mask shown in Figure 1 as well as in line number three of the script example described above. If a network is chosen to be trained without an additional ICA step the network can be fitted with the missing ICA layer at a later stage by using “add_ICA” parameter for training. This is useful for being able to look at the network performance both with and without sparse coding. This also holds when sampling for head direction cells: the original SFA output of a trained network is already sensitive to the direction of the agent, but not clearly tuned to particular directions. Only after adding an additional layer coding for sparseness the model yields the expected head direction plots as depicted in Figure 5.

### Software implementation

**Figure 6:**
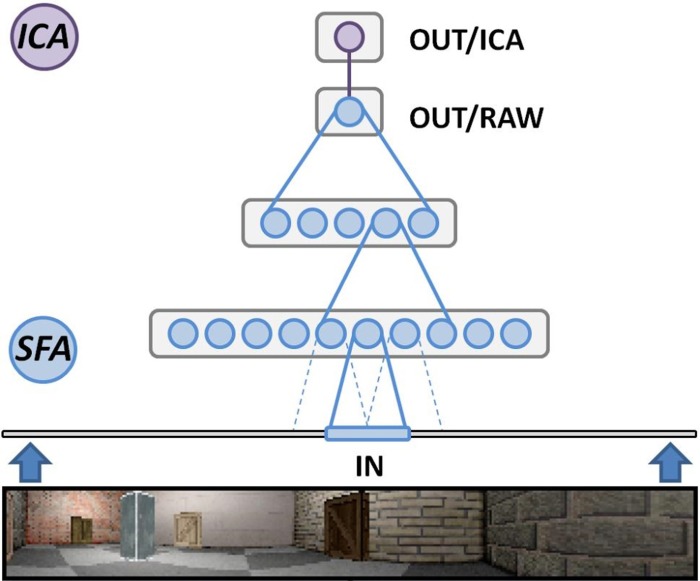
**Hierarchical SFA network**. Schematic overview of the network as it is used in RatLab: three layers of nodes performing the SFA algorithm extract increasingly more abstract information from the visual data stream generated by the software. A final node implementing sparse coding via independent component analysis (ICA) translates the raw SFA output into place cell activity.

When programming in Python the Modular toolkit for Data processing (MDP) library (Berkes and Zito, 2007) provides a complete implementation of SFA, encapsulated within a single node class. The library also contains a package for organizing such nodes in layered networks and is the primary means to perform experiments based on the hierarchical SFA approach. RatLab uses MDP to construct such a hierarchical network consisting of three different SFA layers plus the optional ICA layer described above (Figure 6). The lowest layer works directly on the visual input data and is organized in a two dimensional array of 63 by 9 SFA nodes. Each of these nodes covers a small rectangular area of the incoming video stream and learns to extract simple features based on this section of the input. Neighboring nodes cover overlapping areas of an input frame in order to facilitate feature detection over areas larger than the input window of a single node. Once trained, these nodes share a wide range of properties with the complex cells found in the primary visual cortex (Berkes and Wiskott, 2005). The second SFA layer is organized in the same way and consists of an array of 8 by 2 SFA nodes with overlapping input ranges. These nodes are trained by the output signals of the initial SFA layer extract and more abstract features than the nodes of the first layer. The third layer consists of a single SFA node that integrates the output of all the sixteen nodes in the previous layer. A final node implementing sparse coding works on the output of the highest level SFA node and is required to extract the place field information as it is shown in Figure 4.

As comprehensive as the MDP framework is, however, it also states a barrier of entry for interested parties with only little experience in programming. MDP does also not offer any graphical component to produce the visual data the experiments described in this text are based upon. RatLab utilizes OpenGL and the GLUT utility library [OpenGL, GLUT] to provide platform independent rendering of environments and objects.

In addition, SFA suffers from the “curse of dimensionality”: A high input dimensionality results in a drastically raised requirement of computational resources, i.e., processing power. This is the actual reason to use the hierarchical network of SFA nodes described in Franzius and Wiskott (2007) instead of a single SFA instance. Using this approach any single SFA node within the network has to deal with a significantly lower input dimensionality than a full image frame, resulting in faster training of the overall network. The curse of dimensionality still cannot easily be dismissed, however. When presented originally in Franzius and Wiskott (2007) the network was trained on a hardware cluster, and the MDP library can be used to replicate this parallelized version and add it to RatLab. Natively, however, training in RatLab can optionally be boosted by utilizing an Nvidia graphics card and the freely available Nvidia CUDA tool set [CUDA]. This allows for the use of highly parallel graphics hardware as a co-processor. It is further recommended to use an optimized linear algebra library like MKL^1^ [MKL] to speed up computations that are not relayed to CUDA hardware. In our laboratory the software was developed and used on desktop computers fitted with 16 gb of RAM, an Intel i7 quad core CPU, and a Nvidia GeForce GTX 580 graphics card. On these machines it is possible to produce results as presented in this work in about 50 min. The training of the network takes around 11 min; while most time—about 32 min—is spent sampling the trained networks for the final plots.

As mentioned earlier the RatLab source code primarily consists of four Python core modules. Any automatically generated and most manually written RatLab scripts will thus contain four calls to execute each of these Python programs with the specified parameter selection. Each module implements one step to produce all desired plots from the parameterized description of an experiment:

- ratlab.py: The rendering and recording of the simulation environment as the virtual rodent navigates the environment.
- convert.py: Converting the data from single image frames into a single non-human-readable data file.
- train.py: Training the specified SFA hierarchy with the generated visual data.
- sample.py: Sampling the network over the complete enclosure and generating plots as specified.

These four modules create the following data in this order:

(a.1) The visual data in the form of single color and/or grayscale images.
(a.2) A summary of the experiment including trajectory data and a screenshot of the final state of the experiment to judge how well the enclosure has been traversed by the virtual rat.
(b) The visual data stored in a single, not human readable file.
(c) The trained network stored in a separate file for future use.
(d) All the specified plot images sorted into clearly labeled directories.

After training the actual network is available as a.tsn file, which contains the network as a compressed Python object. Other Python scripts can access this file and extract the stored network via the following lines of code:

import pickle
tsn_file = open(“networkFileName,” ‘rb’)
sfa_network = pickle.load(tsn_file)

The idea of separating the distinct steps of the simulation is to offer the ability to only use the distinct processing step(s) that are currently of interest. In this way a network can be trained once and sampled over various different environments without the need to execute the whole software pipeline multiple times or any additional parameters to specify which stages of the pipeline should be executed and which should not. Trajectory and image data can be re-used as well, for example by applying additional image filters to the visual data and training two networks with the raw and filtered data set—or even compare SFA results with a different model that adheres to the same input/output conventions by replacing the default training module with a custom made one. Another example is using a robot to take pictures for training a network, and then using this network to facilitate place field based navigation on the robot, merely requiring sensory input and thus able to ignore the internally accumulating error of an approach based on motor information and path integration.

## Discussion

This paper presents RatLab, a Python framework to set up and simulate rodent studies examining the hippocampal place code and its reaction to environmental modifications, agent movement statistics, and specific input modulations. RatLab is based on the SFA algorithm which has been shown to produce plausible place fields based on visual training alone, and the toolkit allows for open access to this model without any programming requirements. The software includes all steps from rendering a specified environment with a 320 degree field of view, training the actual network, and automatically producing finished place field plots. The full Python source for RatLab is freely available together with a collection of additional tools, tutorials, and examples for advanced simulation scripts.

In its current version RatLab is being used as part of a larger project based on three concurrent threads: physiological studies in real rats foraging in a virtual reality apparatus, robot experiments with ePuck agents [ePuck], and further software models of the hippocampal spatial code. RatLab takes part in every aspect of these: it can offer predictions and experiments to the physiology laboratory with emphasis on experiments that either cannot be done at all or would require an excessive amount of effort to do in the physical world. With the option to switch to the field of view available to digital cameras, RatLab can simulate experiments to be performed in robots. And finally, trained networks produced by RatLab can be used as modules in a larger software framework and thus be part of a more elaborate architecture to examine the formation and plasticity of the hippocampal place code. Overall, the RatLab toolset serves as an example of how computational models can be useful in facilitating an interdisciplinary approach to neuroscience: experimental studies can be supported and enhanced, while measurement results lead to refinements in the model. The actual work can be performed in a largely independent manner for the involved groups as coordination merely needs to take place when producing new results and/or deciding on follow-up studies. As such, software models like RatLab can offer the advantages of theoretical simulation to experimentalists while at the same time benefiting from real world data to validate the model itself.

**Simulation Steps Summary**

**Figure 7:**
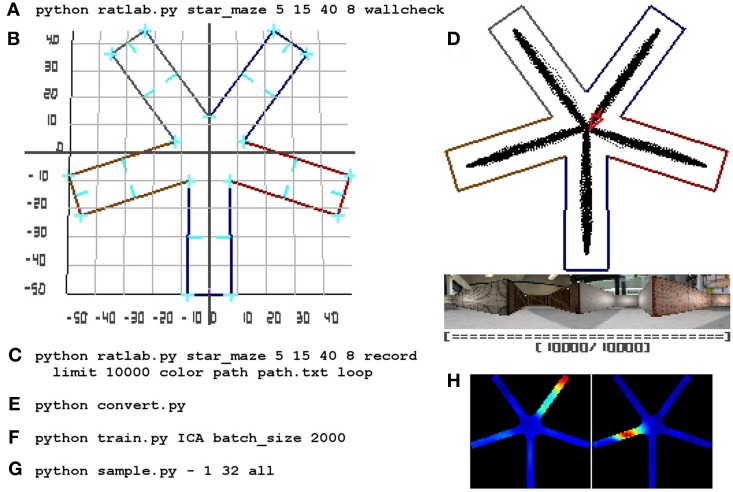
**RatLab reference walkthrough**. Here a complete simulation walkthrough is detailed: **(A)** The initial command to confirm the experimental setup via the *wallcheck* option. **(B)** The generated wallcheck image depicting the correct alignment of all walls and a coordinate raster overlay. The latter is being used as a reference to find the required coordinates for a path that leads the simulated animal successively into each of the arms of the maze. **(C)** The full command to run and record the experiment using a text file storing the coordinates found in the previous step. **(D)** A screenshot of the final state of the simulation showing the area covered by the virtual rodent, the final image of the visual stream, and the now filled progress bar of the simulation. **(E)** The command to collect all image data within a single file. **(F)** Training a new network with the collected data; the network is set to include an additional ICA node at its top and is being trained in batches that fit comfortably in system memory. **(G)** The sampling command to record the slowest 32 signals at each valid coordinate position of the environment. Each location is sampled while looking in eight different directions, as denoted by the “all” parameter; afterwards plots are generated to archive the signal activity in each of the sampled directions as well as an average over all of them. **(H)** Shows three examples of the averaged spatial activity of three cells/signals. The whole process is completed in about 50 min.

For quick reference purposes, Figure 7 summarizes all steps of a complete simulation.

**Online Resources**

The complete RatLab package is available for download under the following address:

http://www.ini.rub.de/research/groups/tns/RatLab.html.en

The website describes the required Python libraries, the necessary steps to install RatLab, and an extensive step by step tutorial of setting up and running a complete RatLab simulation.

### Conflict of interest statement

The authors declare that the research was conducted in the absence of any commercial or financial relationships that could be construed as a potential conflict of interest.

## Footnotes

^1^MKL is free to use for academic purposes.
